# Diagnostic exome sequencing identifies *GLI2* haploinsufficiency and chromosome 20 uniparental disomy in a patient with developmental anomalies

**DOI:** 10.1002/ccr3.1575

**Published:** 2018-05-08

**Authors:** Samin A. Sajan, Zöe Powis, Katherine L. Helbig, Honey Nagakura, Ladonna Immken, Sha Tang, Wendy A. Alcaraz

**Affiliations:** ^1^ Department of Clinical Genomics Ambry Genetics Aliso Viejo CA USA; ^2^ Specially for Children Genetics Austin TX USA; ^3^ Present address: Children's Hospital of Philadelphia Philadelphia PA USA

**Keywords:** developmental anomalies, diagnostic exome sequencing, *GLI2*, uniparental disomy

## Abstract

Clinical diagnostic exome sequencing (DES) is currently infrequently used for detecting uniparental disomy (UPD). We present a patient with a dual diagnosis of *GLI2* haploinsufficiency as well as UPD of chromosome 20, both identified through DES. We therefore recommend routine UPD analysis during DES to identify this genetic aberration.

## 1 INTRODUCTION

Since 2011, clinical diagnostic exome sequencing (DES) has proven cost‐effective and beneficial in providing molecular diagnoses for patients with a broad spectrum of previously undiagnosed genetic diseases and broadening the phenotype of known genetic diseases.^1^

**Figure 1:**
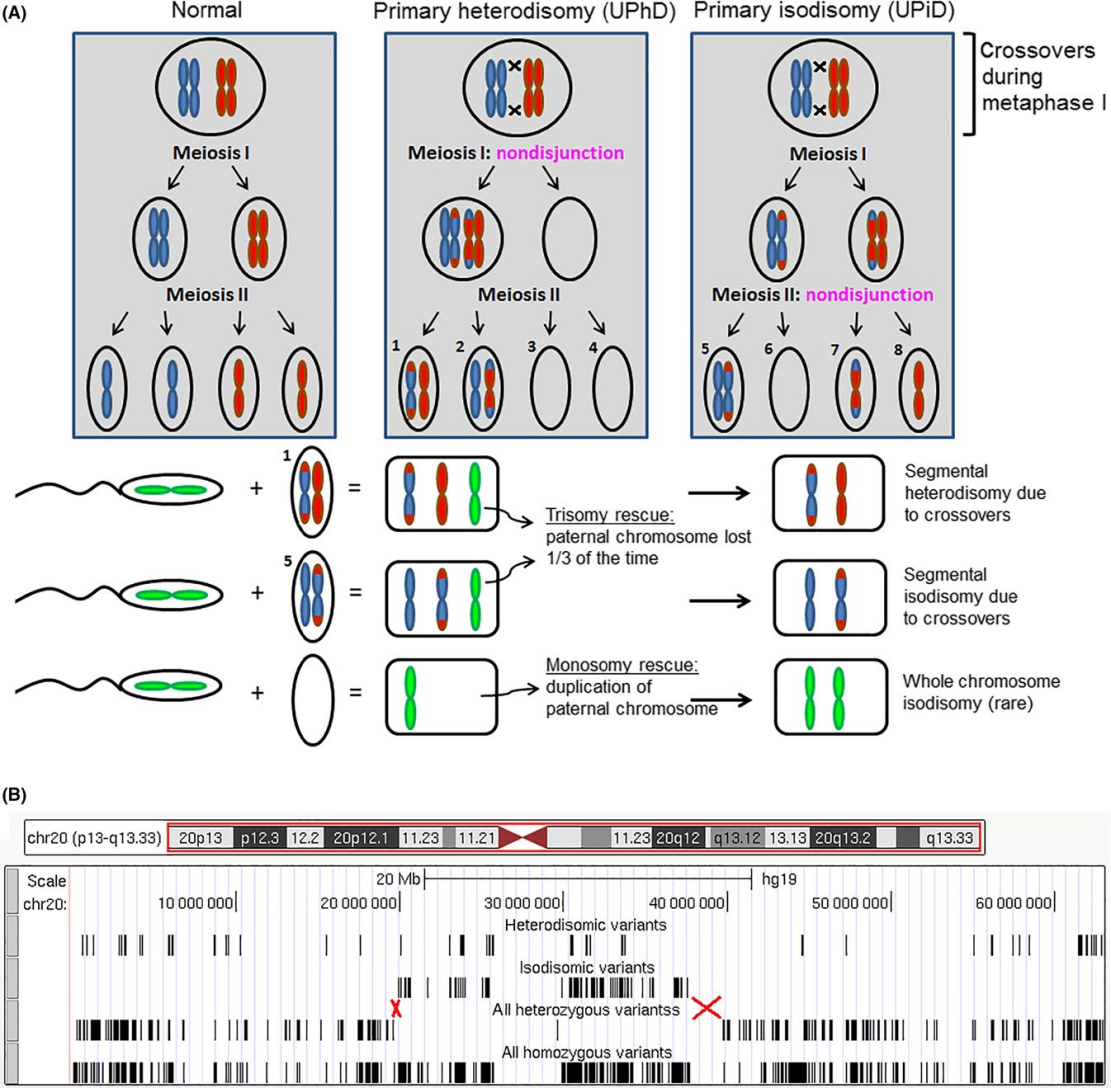
A, Schematic showing normal meiosis as well as nondisjunction events during meiosis I and II in the maternal gamete that results in primary heterodisomy and isodisomy, respectively. The individual maternal gametes are labeled with numbers. Blue and red represent two homologs of a given chromosome, and each single “rod” is a sister chromatid. Meiotic recombination events usually occur prior to the first meiotic division. The lower half shows outcomes of a normal haploid sperm cell fertilizing egg cells containing abnormal chromosomal copies, including a nullisomic egg cell, due to nondisjunction events. B, Distribution of four different types of variants across chromosome 20 in the patient. Each black bar is a single variant. The first row of black bars shows heterodisomic variants which are homozygous variants indicative of maternal uniparental disomy (UPD) but which do not distinguish between isodisomy (UPiD) and heterodisomy (UPhD) and are dispersed randomly across the entire chromosome. The second row of black bars shows isodisomic variants which unambiguously indicate maternal UPiD and are confined to centromere‐proximal regions. The third row of black bars shows all heterozygous variants, and these are confined to centromere‐distal regions. The fourth row of black bars shows all homozygous variants, and they were dispersed across the entire length of the chromosome. Red crosses show regions where meiotic recombination events are predicted to have occurred (chr20:19560664‐19867406 on the p arm and chr20:37554898‐39701015 on the q arm)

Uniparental disomy (UPD) is a recognized clinically relevant finding most commonly diagnosed by methods such as methylation‐specific polymerase chain reaction and methylation analysis.^2^, ^3^ It is important to detect this phenomenon as it can result in imprinting disorders and/or homozygosity of rare recessive mutations.^4^ Multiple mechanisms have been documented that result in UPD.^5^, ^6^ It can occur either as heterodisomy (UPhD) where two different homologous chromosomes are inherited from only one parent or as isodisomy (UPiD) where both homologs are identical due to duplication of one parental homolog (Figure 1A).^5^, ^6^, ^7^ A widely accepted rate of UPD is 1/3500 births, but a higher rate of 6/1057 has recently been observed in a cohort of patients from the Deciphering Developmental Disorders project, suggesting that clinical cohorts have an enhanced rate of UPD.^7^, ^8^, ^9^ Herein, we report a patient presenting with multiple congenital anomalies with a paternally inherited heterozygous truncating mutation in the *GLI family zinc finger 2* (*GLI2*) gene as well as maternal primary UPiD (centromeric isodisomy and distal heterodisomy) of chromosome 20 detected by clinical DES.

## 2 MATERIALS AND METHODS

This study was conducted in accordance with the World Medical Association Declaration of Helsinki. Informed consent was obtained prior to testing. Written informed consent was received from the family for publication of the clinical information.

### 2.1 Exome and sanger sequencing

Exome library preparation, sequencing, bioinformatics, and data analysis were performed as previously described, the exception being the capture reagent which was IDT xGen Exome Research Panel v1.0.^1^, ^10^ Approximately 98% of the patient's and parents' exomes were sequenced at a depth of at least 20X with the mean depth being at least 150X. Bidirectional Sanger sequencing was used to confirm the identified *GLI2* variant.

### 2.2 Variant filtering for exome primary analysis

The father was considered partially affected for the purpose of analysis based on his clinical features. For variant filtering in different genetic models, minor allele frequency cutoffs of 0.1% and 1% were used, respectively, for dominant and recessive models for variants in known Mendelian disease genes. A cutoff of 0.1% and 0.2% was used, respectively, for dominant and recessive models for variants in genes presently not known to cause any Mendelian disease. We examined three models of inheritance postfiltering‐dominant model due to de novo variants as well as variants inherited from the father, recessive model due to homozygous and compound heterozygous variants, and incomplete penetrance model. The latter model contained variants inherited from either parent that were (1) listed in the Human Gene Mutation Database (HGMD) or internally classified as pathogenic or likely pathogenic; (2) loss of function variants such as nonsense, frameshifts, and canonical splice sites; and (3) found in genes that cause imprinted disorders. The patient and both parents were provided with a complementary analysis of secondary findings in the minimum list of genes recommended by the American College of Medical Genetics and Genomics.^11^ There were no reportable secondary findings in anyone.

### 2.3 Variant analysis for UPD identification

Uniparental disomy of chromosome 20 was identified by counting the numbers of uniparentally and biparentally inherited variants. Uniparentally inherited variants were then further subdivided into those specific to isodisomy and those common to both isodisomy and heterodisomy as described.^7^ From a total of 3349 variants on chromosome 20, we selected those that (1) had a quality score of at least 30 from the Genome Analysis Toolkit (GATK), (2) had a coverage of at least 20X, (3) were heterozygous with an alternate allele to reference allele ratio of between 0.2 and 0.8, and (4) were homozygous with an alternate allele to reference allele ratio of no more than 2% if the genotype call was homozygous for the reference allele (unless there was only 1 alternate allele seen) and no less than 98% if the genotype call was homozygous for the alternate allele (unless there was only 1 reference allele seen) in each of the three individuals sequenced. There were 1934 variants that met these criteria (Table S2).

## 3 RESULTS

### 3.1 Clinical case report

The patient was a 3‐year‐old boy of Ecuadorian, English, and German ancestry with overall growth delay, failure to thrive, global developmental delays, sensory feeding issues, ostium secundum‐type atrial septal defect, kyphoscoliosis, 2‐3 toe syndactyly, bilateral cryptorchidism, phimosis, hypotonia, dysmorphic features, and chronic constipation. Prenatal course was complicated by intrauterine growth retardation (IUGR) for which an amniocentesis was performed and demonstrated mosaic trisomy 20 by karyotyping. He was born at 34 weeks with a weight of 2 pounds 4 ounces and a length of 14 inches. Follow‐up postnatal karyotype and chromosome microarray analyses (CMA) were normal, with no copy number abnormalities or large regions of homozygosity (ROH) identified. Additional features included microcephaly, midface hypoplasia, delicate facies, hypotelorism, epicanthal folds, low set ears, small nose, crowded gums (narrow alveolar arches), thin lips, small mouth, bilateral hockey‐stick creases, and broad short neck. He had abnormal strength, mild joint limitation, hunched posture, and wide‐based, irregular gait. His features were thought to resemble his father who was 5′7″ with a history of constitutional delay and 2‐3 toe syndactyly. At the age of 16 years, the father was 4′8″ but then had a growth spurt in high school. The parents reported an early miscarriage. Maternal family history was noncontributory. Consanguinity was not reported.

### 3.2 Analysis of intragenic variants

Exome sequencing identified a previously unreported paternally inherited pathogenic nonsense variant in the *GLI2* gene in the patient, NM_005270.4 c.1648dupC (NP_005261.2 p.R550Pfs*53) expected to result in haploinsufficiency. The variant was confirmed by Sanger sequencing (Figure S1) and was deemed to explain the patient's growth delay, microcephaly, developmental delay, and dysmorphic features including hypotelorism, midface hypoplasia, and small nose, as well as the father's similar facial features. Heterozygous pathogenic alterations in this gene are known to cause a highly variable condition with incomplete penetrance characterized by hypopituitarism, growth hormone deficiency, postaxial polydactyly, and also holoprosencephaly.^12^ There were additional variants in 51 other genes that survived our filtering but were considered irrelevant to the patient's phenotype (Table S1).

### 3.3 Identification of chromosome 20 UPD

Of the 1934 variants on chromosome 20 that satisfied all filtering criteria (Table S2), a subset whose parental genotypes were informative for assessing UPD in the patient showed that the patient lacked biparentally inherited variants (Table 1). Specifically, the patient had inherited variants only from the mother but none from the father (Table 1). Further analysis showed that all of the 140 unambiguously maternal isodisomic variants were localized in the centromere‐spanning region. Based on the genomic positions of the first and last isodisomic variants on the p and q arms, respectively, the minimum size of this region was 17.69 megabases (hg19 coordinates chr20:19867406‐37554898), whereas based on the genomic positions of the last and first heterozygous variants on the p and q arms, respectively, the maximum size of this region was 20.14 megabases (chr20:19560664‐39701015). Altogether, this region contained 544 variants of which 525 (96.5%) had homozygous genotypes in the patient; the 19 heterozygous variants were in the *FRG1BP* pseudogene and were therefore likely false‐positive heterozygous calls. In contrast, centromere‐distal regions had homozygosities of 51.41% (256 of 498 variants on the p arm) and 57.52% (524 of 911 variants on the q arm).

**Table 1:** Counts of informative genotype combinations in the trio indicating chromosome 20 maternal uniparental disomy in the patient [informative genotypes are as described by King et al^7^]

Inheritance	Father's genotype	Mother's genotype	Patient's genotype	Total number of variants with this combination of genotypes in parents	Total number of variants with this combination of genotypes in patient‐parent trio
Biparental	0/1	1/1	0/1	72	0 (0%)
Biparental	1/1	0/0	0/1	57	0 (0%)
Paternal‐isodisomic	0/1	1/1	0/0	161	0 (0%)
Paternal‐isodisomic	0/1	0/0	1/1	449	0 (0%)
Paternal‐heterodisomic or isodisomic (ambiguous)	0/0	1/1	0/0	72	0 (0%)
Paternal‐heterodisomic or isodisomic (ambiguous)	1/1	0/0	1/1	57	0 (0%)
Maternal‐isodisomic	1/1	0/1	0/0	136	11 (8.1%)
Maternal‐isodisomic	0/0	0/1	1/1	518	129 (24.9%)
Maternal‐heterodisomic or isodisomic (ambiguous)	1/1	0/0	0/0	57	57 (100%)
Maternal‐heterodisomic or isodisomic (ambiguous)	0/0	1/1	1/1	72	72 (100%)

The distribution of these variants across the entire chromosome 20 in the patient indicated a meiosis II nondisjunction event during oogenesis resulting in an oocyte containing two identical sister chromatids followed by trisomic rescue (Figure 1A). The confinement of all heterozygous variants to the centromere‐distal regions and of homozygous isodisomic variants to the centromere‐spanning region can be explained by the occurrence of one meiotic recombination event on each chromosome arm just prior to meiosis I. On each arm, the meiotic recombination event is expected to be located between the heterozygous variant closest to the centromere and the isodisomic variant closest to the centromere (Figure 1B). For the p arm, this translated into a 306.7‐kilobase region at chr20:19560664‐19867406 and for the q arm into a 2.15‐megabase region at chr20:37554898‐39701015.

The maternal chromosome 20 UPD was deemed to explain the patient's feeding difficulties, failure to thrive, hypotonia, kyphoscoliosis, and 2‐3 toe syndactyly. These features have previously been reported in other cases of isolated maternal chromosome 20 UPD.^13^

## 4 DISCUSSION

We have presented an unusual case with a dual molecular diagnosis of *GLI2* haploinsufficiency and maternal chromosome 20 UPD, both identified by clinical DES. The UPD in this patient was identified by manual analysis of variants on chromosome 20 due to a previous suspicion of mosaicism of trisomy 20 without any copy number changes. Had the clinician who ordered the DES test for this patient not mentioned this, then the UPD may have been missed by our laboratory. We therefore recommend clinicians to always provide results of previous genetic tests and specifically mention any suspicion of UPD due to, for example, a large region of homozygosity observed only on one particular chromosome via single nucleotide polymorphism (SNP) microarray test without any accompanying copy number changes. Even in the absence of a SNP microarray test, such as in the patient presented here who had a chromosomal microarray test which does not detect homozygous regions, a prenatal karyotype that shows mosaicism for trisomy should be enough to raise suspicion of possible UPD because of the nature of the molecular mechanisms involved. For instance, many UPD's can occur due to rescue of trisomy which can occur due to errors in either meiotic or mitotic cell division; however, depending on whether the rescue occurred early or late after fertilization, the cell lineage, as well as the chromosome involved, there will be a variable proportion of cells that will still appear trisomic during karyotyping via amniocentesis.^14^ The patient described here was karyotyped using cells obtained via amniocentesis, a procedure that samples fetal cells. However, in some cases, patients undergo chorionic villus sampling (CVS), a procedure that collects only placental cells. If karyotyping of placental cells reveals aneuploidy, it is possible that this defect may not be present in the fetus in which case it is known as confined placental mosaicism (CPM). In such cases, therefore, it is necessary to do confirmatory karyotyping on fetal cells via amniocentesis.^15^ Even if amniocentesis reveals a normal karyotype, the possibility of UPD in the fetus cannot be completely ruled out.

Because DES is currently not a standard test for detecting UPD, this patient's result was indicated to be an “uncertain” finding on the clinical report and an independent confirmatory test, such as a trio‐based SNP microarray test, was recommended to the patient. Nevertheless, even if the clinicians who order DES tests fail to mention any suspicions of UPD in their patients as discussed above, it is still feasible for bioinformatics pipelines of clinical laboratories to detect this abnormality for all (or some) chromosomes in all parent‐patient trios based on segregation patterns of variants. Indeed, a recent study of 96 unresolved cases of motoneuron disease and ataxia by exome sequencing revealed one case of complete paternal isodisomy of chromosome 16 resulting in a causative homozygous pathogenic mutation in *FA2H*.^16^ Making UPD analysis a standard part of DES is therefore expected to increase the overall diagnostic yield.

## CONFLICT OF INTERESTS

Samin A. Sajan, Zöe Powis, Wendy Alcaraz, and Sha Tang are employed by and receive a salary from Ambry Genetics, one of whose commercially available tests is exome sequencing. Katherine L. Helbig, Honey Nagakura, and Ladonna Immken have no conflict of interests to declare. No additional funding sources apply.

## AUTHOR CONTRIBUTIONS

SAS: analyzed exome data, drafted, and revised the manuscript. ZP: did background research and revised the manuscript. KLH, ST, and WAA: reviewed exome results and revised the manuscript. HN and LI: involved in patient care.
