# Supplementary material for: Diagnostic exome sequencing identifies GLI2 haploinsufficiency and chromosome 20 uniparental disomy in a patient with developmental anomalies
Source: Clin Case Rep. 2018 May 8;6(7):1208–13. doi: 10.1002/ccr3.1575 (PMC6028413; doi:10.1002/ccr3.1575)
Supplement: Supplementary file 4 [file CCR3-6-1208-s004.docx]

Figure S1. Sanger sequence-based confirmation of the GLI2 frameshift variant. Bi-directional Sanger sequencing chromatograms confirming the GLI2 mutation c.1648dupC (p.R550Pfs*53) in the father, mother, and proband. Colors of the reverse strand have been changed to reflect those on the forward strand
