# Supplementary material for: Diagnostic exome sequencing identifies GLI2 haploinsufficiency and chromosome 20 uniparental disomy in a patient with developmental anomalies
Source: Clin Case Rep. 2018 May 8;6(7):1208–13. doi: 10.1002/ccr3.1575 (PMC6028413; doi:10.1002/ccr3.1575)
Supplement: Supplementary file 5 [file CCR3-6-1208-s005.docx]

Table S1. List of all genes and variants that passed exome filtering criteria but which were not considered to be relevant to the proband’s phenotype.
