# Supplementary material for: Diagnostic exome sequencing identifies GLI2 haploinsufficiency and chromosome 20 uniparental disomy in a patient with developmental anomalies
Source: Clin Case Rep. 2018 May 8;6(7):1208–13. doi: 10.1002/ccr3.1575 (PMC6028413; doi:10.1002/ccr3.1575)
Supplement: Supplementary file 6 [file CCR3-6-1208-s006.docx]

Table S2. List of all 1,934 variants identified by exome sequencing in the proband and both parents after applying the filters described in Methods for determining uniparental disomy.
